# Establishment and verification a nomogram for predicting portal vein thrombosis presence among admitted cirrhotic patients

**DOI:** 10.3389/fmed.2022.1021899

**Published:** 2023-01-06

**Authors:** Guang-hua Liu, Ping Lei, Chu-shu Liao, Jing Li, Jiang-wen Long, Xi-sha Huan, Jie Chen

**Affiliations:** ^1^Department of Blood Transfusion, Hunan Provincial People's Hospital, The First Affiliated Hospital of Hunan Normal University, Changsha, China; ^2^Laboratory of Hematology, Hunan Provincial People's Hospital, The First Affiliated Hospital of Hunan Normal University, Changsha, China; ^3^Department of Clinical Laboratory, Hunan Provincial People's Hospital, The First Affiliated Hospital of Hunan Normal University, Changsha, China

**Keywords:** portal vein, thrombosis, cirrhosis, risk factors, nomogram

## Abstract

**Background:**

Portal vein thrombosis (PVT) is an increasingly recognized complication of cirrhosis and possibly associated with mortality. This study aims to evaluate provoking factors for PVT, then establish a concise and efficient nomogram for predicting PVT presence among admitted cirrhotic patients.

**Materials and methods:**

All cirrhotic patients admitted in Hunan Provincial People's Hospital between January 2010 and September 2020 were retrospectively reviewed, the clinical and laboratory data were collected. Multivariate logistic regression analysis and the least absolute shrinkage and selection operator regression method were used for screening the independent predictors and constructing the nomogram. The calibration curve was plotted to evaluate the consistent degree between observed outcomes and predicted probabilities. The area under the receiver operating characteristics curve was used to assess the discriminant performance. The decision curve analysis (DCA) was carried out to evaluate the benefits of nomogram.

**Results:**

A total of 4,479 patients with cirrhosis were enrolled and 281 patients were identified with PVT. Smoking history, splenomegaly, esophagogastric varices, surgical history, red blood cell transfusion, and D-dimer were independent risk factors for PVT in cirrhosis. A nomogram was established with a good discrimination capacity and predictive efficiency with an the area under the curve (AUC) of 0.704 (95% CI: 0.664–0.745) in the training set and 0.685 (95% CI: 0.615–0.754) in the validation set. DCA suggested the net benefit of nomogram had a superior risk threshold probability.

**Conclusion:**

A concise and efficient nomogram was established with good performance, which may aid clinical decision making and guide best treatment measures.

## Introduction

Portal vein thrombosis (PVT) is defined as the presence of a thrombus in the lumen of the main portal vein that can extend into intrahepatic or extrahepatic venous branches. Clinical features of PVT are heterogeneous and non-specific, mainly associated with the site and extension of its obstruction in the portal venous system, resulting in a variety of clinical consequences ranging from an absence of symptoms to liver decompensation, intestinal ischemia and worsening portal hypertension (1–3). PVT is a critical and frequent complication of liver cirrhosis (4, 5), which deteriorates liver function and increases the risk of bleeding (6). It has been reported that PVT may have a relationship with mortality (7). The prevalence and incidence of PVT vary due to heterogeneous diagnostic approach, as the frequency of liver imaging increases, it is increasingly identified in cirrhotic patients with an estimated annual incidence ranging from 4.6 to 26% (8).

The pathogenesis of PVT in cirrhosis is probably multifactorial, several risk factors associated with the occurrence and progression of PVT have been proposed: hypercoagulability, altered portal venous blood flow and local portal vein alterations (4). Besides, it has been also proposed that beta-blockers, hepatic encephalopathy, prothrombin time, and baseline esophageal varices were risk factors compared with patients without PVT (9, 10). Additionally, invasive operations such as splenectomy (11), abdominal surgery and endoscopic therapy for esophageal varices have been incriminated in PVT development by reason of venous injury and altering portal venous flow (12).

Identification of risk factors that predispose to PVT presence and early assessment the presence among admitted cirrhotic patients is crucial for the diagnosis, prognosis and appropriate management of these patients. Various nomogram models have been proposed for prediction of PVT in patients with liver cirrhosis (11, 13, 14). However, these models are either small sample size or study subjects limiting liver cirrhosis with splenectomy. Thus, a comprehensive predictive scoring system is urgently required for determining the risk of PVT. The aim of the current study is to evaluate the underlying provoking factors for PVT presence in a large cohort of admitted cirrhotic patients, then establish and verify a concise and efficient prediction model to help clinicians make timely, individualized clinical decisions.

## Materials and methods

### Patients and data collection

All patients with cirrhosis identified by clinical records of Hunan Provincial People's Hospital between January 2010 and September 2020 were retrospectively reviewed. The diagnosis of cirrhosis was in accordance with the Chinese guidelines on the management of cirrhosis (15). PVT was confirmed by Doppler ultrasound, magnetic resonance imaging and computerized tomography during hospitalization. The exclusion criteria were as follows: (1) incomplete data; (2) age < 18 years old; (3) concomitant with malignant tumors, hematological diseases and autoimmune system diseases; (4) have received antiplatelet or anticoagulant drugs treatment. (5) PVT occurred before transfusion. Patients with readmission and multiple hospitalizations, only the first admission for all patients was included. Patients with cirrhosis were randomly divided into the training set and validation set in a proportion of 3:1 to develop and verify the nomogram. According to the presence or absence of PVT, the patients in the training set were divided into PVT group and non-PVT group. This study was approved by Hunan Provincial People's Hospital Medical Ethics Committee. All procedures were performed in accordance with the ethical standards of the institutional research committee.

The clinical and laboratory data were collected including age, gender, smoking history, complications of cirrhosis (including splenomegaly, portal hypertension, esophagogastric varices, gastrointestinal bleeding, ascites and encephalopathy), the cause of the admission, the etiologies of cirrhosis (including hepatitis, alcoholic, autoimmune, NAFLD/NASH, drugs, genetic factors and parasitic infections), surgical history (including splenectomy, gastrectomy, cholecystectomy), surgical history within 2 years before the diagnosis of PVT were included (16, 17), hypertension, diabetes, heart disease, sepsis, Child pugh classification of cirrhosis, white blood cell count (WBC), hemoglobin (Hb), hematocrit (HCT), platelet count (PLT), prothrombin time (PT), activated partial thromboplastin time (APTT), international normalized ratio (INR), D-dimer (DDi), total bilirubin (TB), albumin (ALB), alanine transaminase (ALT), and aspartate aminotransferase (AST).

### Statistical methods

All statistical analysis were performed using SPSS (version 23.0) and R (version 4.2.1) software. Statistical analysis categorical variables were presented as number (percentage) and continuous variables as median [interquartile ranges (IQR)]. The chi-square test was used to compare categorical variables and the Mann-Whitney *U*-test was used to compare continuous variables. The least absolute shrinkage and selection operator (LASSO) regression method was used to select the predictive factors of PVT in patients with cirrhosis at the optimal λ selected by cross validation.

The independent predictors chosen in the LASSO regression method were further performed by multivariate logistic regression analysis and the odds ratios (OR) with a 95% CI were calculated. The independent risk factors associated with PVT obtained from the multivariate logistic regression were used for constructing the predictive model. The calibration curve was plotted to evaluate the consistent degree between observed outcomes and predicted probabilities. The area under the receiver operating characteristics (ROC) curve was used to assess the discriminant performance of the nomogram in the training set and validation set with values closer to 1 indicating higher discrimination ability. Since the area under the curve (AUC) was not enough for decision-making, DCA was carried out to evaluate the clinical usefulness and net benefits of the nomogram at different threshold probabilities. All tests were two-tailed, and *P*-values of <0.05 were considered statistically significant.

## Results

### Patients' characteristics

A total of 4,479 patients with cirrhosis were enrolled in this study and 281 (6.3%) patients were identified with PVT. Infection was the most common reason for hospitalization in cirrhotic patients in this study, followed by abdominal discomfort. The most frequent etiologies of cirrhosis were hepatitis (61.6%) and alcohol (22.4%). 3,360 patients were randomly assigned to the training set and 1,119 patients randomly assigned to the validation set. The rate of PVT in the training set and validation set was 6.0 and 7.0%, respectively (Table 1).

**Table 1:** Demographics, clinical and laboratory data of patients with cirrhosis.

**Variable**	**All patients**	**Training set**	**Validation set**
	***n* = 4,479**	***n* = 3,360**	***n* = 1,119**
Age (year)	55 (47–64)	55 (47–64)	56 (47–65)
Sex (male/female)	3,082/1,397	2,315/1,045	767/352
Smoking history	424 (9.5%)	303 (9.0%)	121 (10.8%)
Cause of admission			
Gastrointestinal bleeding	587 (13.1%)	444 (13.2%)	143 (12.8%)
Ascites	892 (19.9%)	671 (20.0%)	221 (19.7%)
Jaundice	382 (8.5%)	283 (8.4%)	99 (8.8%)
Liver dysfunction	236 (5.3%)	179 (5.3%)	57 (5.1%)
Infection	1,117 (24.9%)	834 (24.8%)	283 (25.3%)
Abdominal discomfort	993 (22.2%)	745 (22.2%)	248 (22.2%)
Other	272 (6.1%)	204 (6.1%)	68 (6.1%)
Etiology			
Hepatitis	2,761 (61.6%)	2,096 (62.4%)	665 (59.4%)
Alcoholic	1,003 (22.4%)	717 (21.3%)	286 (25.6%)
Autoimmune	124 (2.8%)	95 (2.8%)	29 (2.6%)
Other	591 (13.2%)	452 (13.5%)	139 (12.4%)
Complications			
Splenomegaly	2,895 (64.6%)	2,177 (64.8%)	718 (64.2%)
Portal hypertension	1,868 (41.7%)	1,410 (42.0%)	458 (40.9%)
Esophagogastric varices	1,502 (33.5%)	1,139 (33.9%)	363 (32.4%)
Gastrointestinal bleeding	654 (14.6%)	496 (14.8%)	158 (14.1%)
Ascites	1,538 (34.3%)	1,160 (34.5%)	378 (33.8%)
Encephalopathy	239 (5.3%)	180 (5.4%)	59 (5.3%)
Comorbidities			
Hypertension	718 (16.0%)	533 (15.9%)	185 (16.5%)
Diabetes	674 (15.0%)	509 (15.1%)	165 (14.7%)
Heart disease	229 (5.1%)	182 (5.4%)	47 (4.2%)
Renal failure	25 (0.6%)	22 (0.7%)	3 (0.3%)
Surgical history	472 (10.5%)	353 (10.5%)	119 (10.6%)
Sepsis	79 (1.8%)	61 (1.8%)	18 (1.6%)
Child-pugh classification			
A	1,560 (34.8%)	1,149 (34.2%)	411 (36.7%)
B	1,980 (44.2%)	1,500 (44.6%)	480 (42.9%)
C	939 (21.0%)	711 (21.2%)	228 (20.4%)
RBC transfusion	387 (8.6%)	302 (9.0%)	85 (7.6%)
WBC (× 10^9^/L)	4.90 (3.44–6.85)	4.89 (3.44–6.85)	4.91 (3.43–6.85)
Hb (g/L)	110 (90–127)	110 (90–127)	110 (90–127)
HCT (%)	33.2 (27.5–38.2)	33.1 (27.5–38.1)	33.5 (27.6–38.4)
PLT (× 10^9^/L)	97 (60–151)	97 (60–151)	98 (60–154)
PT (s)	13.5 (11.9–16.0)	13.5 (11.9–16.0)	13.5 (12.0–15.9)
INR	1.18 (1.05–1.39)	1.18 (1.05–1.39)	1.18 (1.05–1.40)
APTT (s)	38.0 (31.3–47.5)	38.1 (31.4–47.5)	37.8 (30.9–47.6)
DDi (mg/L)	1.07 (0.41–2.84)	1.06 (0.41–2.84)	1.08 (0.40–2.84)
TB (μmol/L)	27.5 (16.5–57.0)	27.6 (16.7–56.6)	27.3 (16.2–57.1)
ALB (g/L)	31.5 (27.4–35.9)	31.4 (27.3–35.7)	31.8 (27.5–36.6)
ALT (U/L)	41.4 (23.9–89.8)	41.9 (23.8–89.1)	40.5 (24.3–93.9)
AST (U/L)	59.2 (34.1–116.2)	59.7 (34.1–115.3)	57.2 (34.2–118.4)
PVT	281 (6.3%)	203 (6.0%)	78 (7.0%)

RBC transfusion, red blood cell transfusion; WBC, white blood cell count; Hb, hemoglobin; Hct, Hematokrit; PT, prothrombin time; APTT, activated partial thromboplastin time; INR, international normalized ratio; DDi, D-dimer; TB, total bilirubin; ALB, albumin; ALT, alanine transaminase; AST, aspartate aminotransferase; PVT, portal vein thrombosis.

For the training set, 3,157 and 203 patients were classified into the non-PVT and PVT group, respectively. Infection, gastrointestinal bleeding and ascites were the three major reasons for hospitalization in cirrhotic patients with PVT. Hepatitis (including hepatitis B and hepatitis C) was the most frequent etiology in the non-PVT and PVT group. However, there was no difference in etiology between the two groups. The PVT group had a higher rate of smoking history compared with non-PVT group. A higher incidence of complications was observed in PVT group, except ascites. The proportions of surgical history and RBC transfusion in the PVT group were higher than those in the non-PVT group. The levels of PT, INR, and INR were significantly higher in the PVT group compared with non-PVT group, while the levels of WBC, Hb, HCT, ALB, ALT, and AST were significantly lower in the PVT group (Table 2).

**Table 2:** Comparison of demographics, clinical and laboratory data between the non-PVT group and PVT group in the training set.

**Variable**	**non-PVT**	**PVT**	***P***
	***n* = 3,157**	***n* = 203**	
Age (year)	55 (47–64)	56 (50–64)	0.086
Sex (male/female)	2,172/985	143/60	0.624
Smoking history	261 (8.3%)	42 (20.7%)	<0.001
Cause of admission			0.003
Gastrointestinal bleeding	401 (12.7%)	43 (21.2%)	
Ascites	628 (19.9%)	43 (21.2%)	
Jaundice	272 (8.6%)	11 (5.4%)	
Dysfunction of liver	171 (5.4%)	8 (3.9%)	
Infection	781 (24.7%)	53 (26.1%)	
Abdominal discomfort	715 (22.6%)	30 (14.8%)	
Other	189 (6.0%)	15 (7.4%)	
Etiology			0.763
Hepatitis	1,965 (62.2%)	131 (64.5%)	
Alcoholic	673 (21.3%)	44 (21.7%)	
Autoimmune	91 (2.9%)	4 (2.0%)	
Other	428 (13.6%)	24 (11.8%)	
Complications			
Splenomegaly	2,012 (63.7%)	165 (81.3%)	<0.001
Portal_hypertension	1,281 (40.6%)	129 (63.5%)	<0.001
Esophagogastric varices	1,023 (32.4%)	116 (57.1%)	<0.001
Gastrointestinal_bleeding	449 (14.2%)	47 (23.2%)	0.001
Ascites	1,081 (34.2%)	79 (38.9%)	0.174
Encephalopathy	163 (5.2%)	17 (8.4%)	0.049
Comorbidities			
Hypertension	504 (16.0%)	29 (14.3%)	0.526
Diabetes	469 (14.9%)	40 (19.7%)	0.062
Heart disease	173 (5.5%)	9 (4.4%)	0.523
Renal failure	21 (0.7%)	1 (0.5%)	0.768
Surgical history	307 (9.7%)	46 (22.7%)	<0.001
Sepsis	59 (1.9%)	2 (1.0%)	0.361
Child-pugh classification			0.416
A	1,088 (34.5%)	61 (30.0%)	
B	1,402 (44.4%)	98 (48.3%)	
C	667 (21.1%)	44 (21.7%)	
RBC transfusion	264 (8.4%)	38 (18.7%)	<0.001
WBC (× 10^9^/L)	4.91 (3.47–6.88)	4.38 (3.09–6.50)	0.040
Hb (g/L)	111 (90–127)	100 (80–116)	<0.001
HCT (%)	33.3 (27.6–38.3)	31.1 (25.3–35.1)	<0.001
PLT (× 10^9^/L)	97 (60–151)	91 (49–145)	0.114
PT (s)	13.5 (11.9–16.0)	13.9 (12.3–16.6)	0.044
INR	1.18 (1.04–1.38)	1.21 (1.08–1.44)	0.036
APTT (s)	38.0 (31.4–47.3)	39.1 (31.0–49.3)	0.473
DDi (mg/L)	1.03 (0.40–2.77)	1.76 (0.54–4.73)	<0.001
TB (μmol/L)	27.7 (16.7–58.5)	27.4 (16.9–46.4)	0.645
ALB (g/L)	31.5 (27.4–35.8)	29.6 (25.9–34.6)	0.003
ALT (U/L)	43.1 (24.1–91.4)	29.5 (19.1–52.3)	<0.001
AST (U/L)	61.0 (34.6–117.5)	42.6 (28.9–82.4)	<0.001

RBC transfusion, red blood cell transfusion; WBC, white blood cell count; Hb, hemoglobin; Hct, Hematokrit; PT, prothrombin time; APTT, activated partial thromboplastin time; INR, international normalized ratio; DDi, D-dimer; TB, total bilirubin; ALB, albumin; ALT, alanine transaminase; AST, aspartate aminotransferase.

### Nomogram establishment

**Figure 1:**
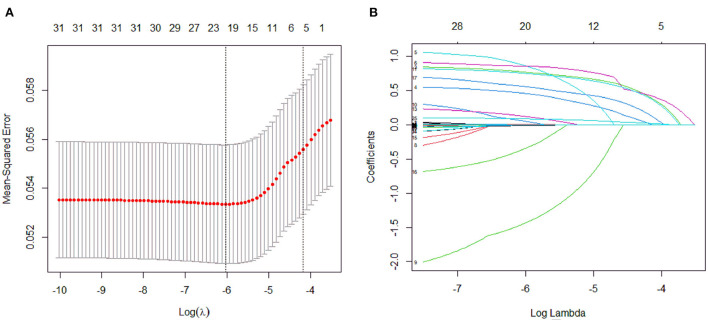
Variables selection and coefficient estimation by LASSO regression analysis. **(A)** Ten-fold cross-validation was performed to select the optimal λ with the minimized binomial deviance. Dotted vertical lines were drawn at the optimal values by using the minimum criteria and the 1 SE of the minimum criteria. **(B)** LASSO coefficient profiles of the 31 variables.

**Figure 2:**
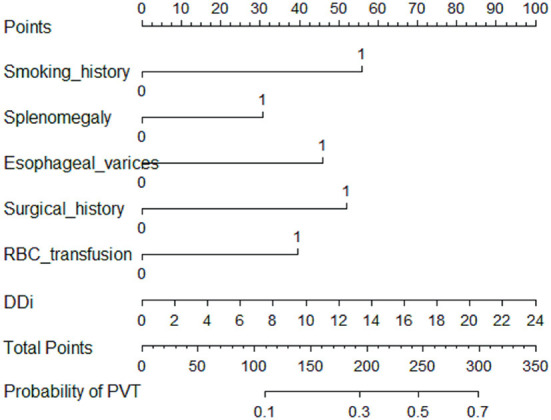
Nomogram for predicting PVT in patients with cirrhosis. In the nomogram, a corresponding score was assigned to each variable on the point scale axis. The sum of all variables located on the total points axis, and then the probability of PVT in patients with cirrhosis was calculated.

A total of 31 variables (Table 2) were incorporated into the LASSO regression analysis (Figures 1, 2), and six variables with optimal lambda (λ = 0.0155) were selected: smoking history, splenomegaly, esophagogastric varices, surgical history, RBC transfusion, and DDi (Table 3). The six variables chosen in the LASSO regression analysis were further brought into multivariate logistic regression. The *P*-values of these six variables, as well as the calculated relative risks were listed in Table 4. These six variables certified as independent risk factors were used to construct the nomogram for predicting PVT in the training set (Figure 2). By assigning a corresponding score for each variable on the point scale axis and placing the sum scores of these six variables on the total point scale axis, the probability of PVT in patients with cirrhosis can be easily calculated.

**Table 3:** Least absolute shrinkage and selection operator regression coefficient table.

**Variable**	**Coefficients**	**Lambda.1se**
Smoking history	0.0276	0.0155
Splenomegaly	0.0004	
Esophagogastric varices	0.0258	
Surgical history	0.0258	
RBC transfusion	0.0095	
DDi	0.0015	

RBC transfusion, red blood cell transfusion; DDi, D-dimer.

**Table 4:** Multivariate logistic regression analysis of the selected variables in the training set.

**Variable**	**Coefficients**	**SE**	**OR**	**95% CI**	***P***
Smoking history	0.899	0.193	2.456	1.682–3.588	<0.001
Splenomegaly	0.495	0.196	1.641	1.118–2.409	0.011
Esophagogastric varices	0.736	0.154	2.087	1.542–2.825	<0.001
Surgical history	0.833	0.190	2.301	1.585–3.342	<0.001
RBC transfusion	0.638	0.201	1.892	1.277–2.804	0.001
DDi	0.067	0.018	1.069	1.032–1.108	<0.001

RBC transfusion, red blood cell transfusion; DDi, D-dimer.

### Nomogram validation

**Figure 3:**
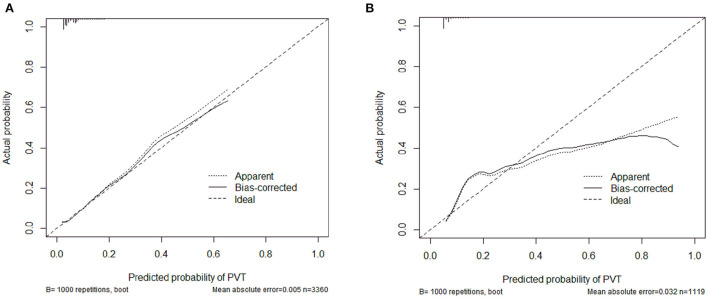
Calibration curves of the nomogram in the training set **(A)** and validation set **(B)**.

**Figure 4:**
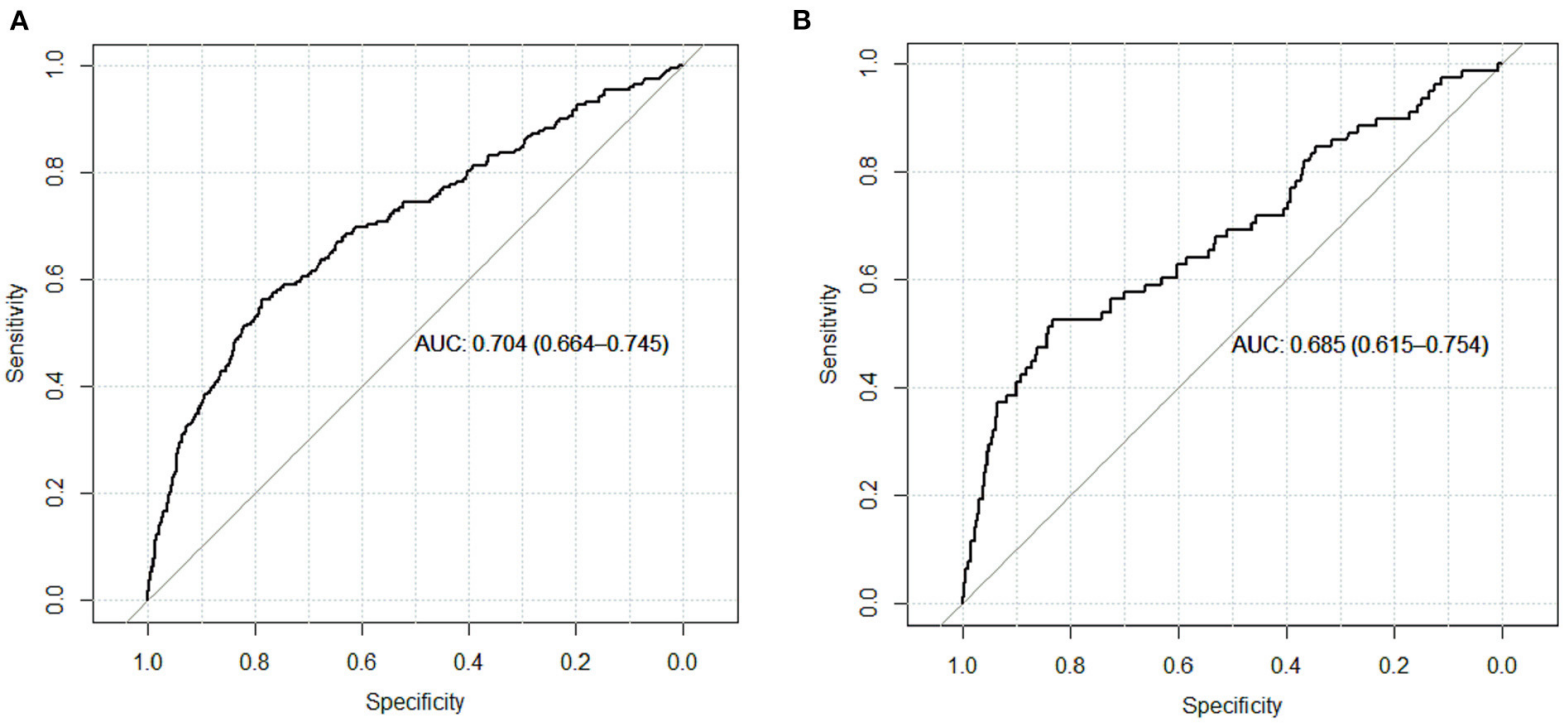
ROC curves for PVT prediction model in the training set **(A)** and validation set **(B)**.

**Figure 5:**
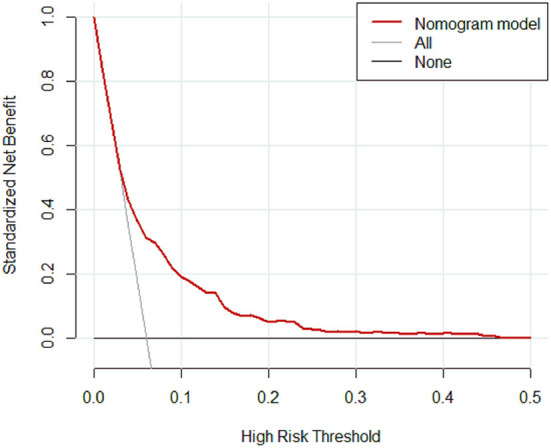
Decision curve analysis for the nomogram.

The calibration curve of the nomogram was calibrated by the bootstrap resampling method with 1,000 repetitions and showed a good consistency between observation and prediction in the training set (Figure 3A) and validation set (Figure 3B). The nomogram achieved an AUC of 0.704 (95% CI: 0.664–0.745) in the training set (Figure 4A) and 0.685 (95% CI: 0.615–0.754) in the validation set (Figure 4B), indicating a good discrimination capacity and predictive efficiency. In addition, DCA suggested that the net benefit of the nomogram had a superior risk threshold probability compared with the baseline (Figure 5), which indicated a good clinical usefulness. The nomogram was established with good performance for predicting PVT among admitted cirrhotic patients.

## Discussion

In the present study, the results shed light on the risk factors of PVT among admitted patients with cirrhosis. Smoking, splenomegaly, esophageal varices, RBC transfusion, DDI, surgical history were identified as risk factors, and independently associated with PVT. A concise and efficient nomogram was established according to these six factors. The nomogram achieved a good consistency between observation and prediction in the training set, and showed a good discrimination capacity and predictive efficiency.

It is well-established that smoking increases blood coagulability, modulates the levels of coagulation factors (18) and impairs endothelial function (19), with a thrombotic potential. Accumulated data shows that smoking is consistently associated with higher venous thromboembolism (VTE) and splanchnic vein thrombosis (20–22). Consistent with previous studies, smoking was confirmed to be an independent risk factor of PVT. Splenomegaly is a concomitant manifestation of many diseases such as chronic liver disease, infections and hematologic diseases (23). Study shows that patients without cirrhosis with PVT have significant splenomegaly (24), it is considered one of the main predisposing factors of PVT after pure laparoscopic splenectomy (23). Consistently, splenomegaly was identified as an independent risk factor of PVT. A growing body of data suggests that an association between PVT development and esophageal varices. The presence of esophageal varices is significantly correlated with a higher rate of PVT, demonstrating esophageal varices may be an important part evaluating the risk of PVT (25–27). In this study, esophageal varices was identified as a significant PVT predictor in cirrhotic patients. Attention about RBC transfusion association with thrombosis has increased dramatically. It has been reported that RBC transfusion is associated with an increased risk of VTE (28, 29). Several possible explanations for transfusion-triggered VTE, RBC transfusion augments blood viscosity, increases platelet interactions and modulates the inflammatory cascades (30). Similarly, RBC transfusion was confirmed to have a connection with PVT after multivariate analysis. DDi is a small peptide fragments of early thrombosis in the role of fibrinogen degradation and plasmin formation, the diagnosis value of DDi in thrombotic events has been well-established. Strong evidence shows that cirrhotic patients with PVT has a significantly higher serum level of DDi compared to patients without PVT (12). DDi can predict the incidence of PVT and may be regarded as a stable and good indicator (31). In accordance with these results, DDi levels were high in cirrhotic patients with PVT, an association between plasma DDi level and an increased risk of PVT was proposed in this study. In contrast to our results, DDi was not an independent predictive factor in a prospective study (8), there are several reasons may explain: in the prospective study, most patients included were Child-Pugh A, the risk factors for PVT in Child-Pugh B and C patients were not known. The sample size was small with 369 patients included, and the most frequent etiologies of cirrhosis were HCV and alcohol. While in our study, most patients included were Child-Pugh B and C, the sample size was larger with 4,479 patients included; and the most frequent etiologies of cirrhosis were hepatitis (hepatitis B and hepatitis C), each of which may influence the result.

An increasing body of evidence suggests that invasive surgery was associated with an increased risk of PVT, including hepatectomy, splenectomy and bariatric surgery, PVT is a frequent complication after operation (12, 32, 33). In consistency with these studies, surgical history is a hazard factor of PVT in patients with cirrhosis.

Recently, a practical nomogram based on systemic inflammatory markers for predicting PVT in patients with liver cirrhosis was established (13). Four hundred and seventy-eighth patients with cirrhosis were included in their study, six independent predictors including neutrophil-to-lymphocyte ratio, platelet-to-lymphocyte ratio, endoscopic ligation, DDi, splenic vein diameter, splenectomy, and autoimmune liver disease were involved in the nomogram. The ROC curve of the model was 0.891 (95% CI: 0.862–0.919), the calibration plots for bootstrap resampling validation showed good consistency. Another nomogram model for prediction of PVT in patients with liver cirrhosis after splenectomy was developed, 315 patients with cirrhosis were included, four predictors of PVT including portal vein diameter, splenic vein diameter, body mass index and platelet count was involved, the nomogram had good predictive efficiency with an AUROC of 0.887 (0.856 in internal validation and 0.796 in in-dependent validation) (11). Similarly, a nomogram for predicting PVT after splenectomy in patients with hepatitis B cirrhosis was constructed, 180 patients with cirrhosis were included in their study, portal vein diameter, splenic vein diameter and PLT addition were involved in the nomogram. with an AUROC of 0.880 in internal validation (95% CI: 0.818–0.942) and 0.873 in external validation (95% CI: 0.785–0.960) (14). In their research, the sample size was small, two of which were mainly focused on liver cirrhosis after splenectomy. Clinically, the fact is that fewer patients underwent splenectomy in cirrhosis, unless the huge spleen affects life, or the leukocytes and platelets are extremely low. In our research, 4,479 cirrhotic patients were included in our study, a nomogram was well-designed based on six easily obtainable, inexpensive clinical and laboratory variables (smoking, splenomegaly, esophageal varices, RBC transfusion, DDi, and surgical history). The AUC was 0.704 (95% CI: 0.664–0.745) and 0.685 (95% CI: 0.615–0.754) in the training set and validation set, respectively. The subjects are not limited to cirrhosis with splenectomy, splenectomy was only as a factor in this study. Besides, RBC transfusion was also taken into account in the model compared to previous studies. Although the AUC maybe not nice, the sample size was larger and it may be more conclusive.

Some inherent limitations existed in the current study. Firstly, this is a cross-sectional study, all the data and results were obtained from a single center. Therefore, further multicenter studies and prospective randomized controlled trials are required to better understand the predictors of PVT in patients with cirrhosis. Second, like other multivariate analysis, some potential risk factors might be missed, not all potential risk factors like systemic inflammatory markers were included in the study. Thirdly, other sites of VTE such as deep vein thrombosis and pulmonary embolism were not assessed in this study. Fourthly, not all patients meet the protocol for PVT screening receiving ultrasound every 6 months, which can affect the results to some extent. Fifthly, the lack of follow-up data precludes from performing Cox modeling with competitive risk for liver transplant and death, which is the recommend type of analysis for this type of study. Moreover, there were no details about the impact of PVT presence and/or anticoagulation treatment on survival and progression of cirrhotic patients, further prospective studies are needed.

## Conclusion

An accurate nomogram for predicting PVT presence among admitted cirrhotic patients was well-developed, it may be of great significance for clinicians to quickly assess the risk of PVT and make timely, more-targeted decisions.

## Data availability statement

The datasets generated for the current study are not publicly available due to data protection but are available from the corresponding author on reasonable request.

## Ethics statement

This study protocol was approved by Hunan Provincial People's Hospital Medical Ethics Committee.

## Author contributions

JC and G-hL developed the study design. G-hL wrote the original draft. JC reviewed the manuscript. PL, C-sL, JL, J-wL, and X-sH were responsible for data and methodology. All authors approved the final version to be published.
